# *Helicobacter pylori* from Gastric Cancer and Duodenal Ulcer Show Same Phylogeographic Origin in the Andean Region in Colombia

**DOI:** 10.1371/journal.pone.0105392

**Published:** 2014-08-14

**Authors:** Seiji Shiota, Rumiko Suzuki, Yuichi Matsuo, Muhammad Miftahussurur, Trang Thu Huyen Tran, Tran Thanh Binh, Yoshio Yamaoka

**Affiliations:** 1 Department of Environmental and Preventive Medicine, Oita University Faculty of Medicine, Yufu, Japan; 2 Department of Medicine-Gastroenterology, Michael E. DeBakey Veterans Affairs Medical Center and Baylor College of Medicine, Houston, Texas, United States of America; National Cancer Center, Japan

## Abstract

**Background:**

A recent report has shown that the phylogenetic origin of *Helicobacter pylori* based on multi-locus sequence typing (MLST) was significantly associated with the severity of gastritis in Colombia. However, the potential relationship between phylogenetic origin and clinical outcomes was not examined in that study. If the phylogenetic origin rather than virulence factors were truly associated with clinical outcomes, identifying a population at high risk for gastric cancer in Colombia would be relatively straightforward. In this study, we examined the phylogenetic origins of strains from gastric cancer and duodenal ulcer patients living in Bogota, Colombia.

**Methods:**

We included 35 gastric cancer patients and 31 duodenal ulcer patients, which are considered the variant outcomes. The genotypes of *cagA* and *vacA* were determined by polymerase chain reaction. The genealogy of these Colombian strains was analyzed by MLST. Bacterial population structure was analyzed using STRUCTURE software.

**Results:**

*H. pylori* strains from gastric cancer and duodenal ulcer patients were scattered in the phylogenetic tree; thus, we did not detect any difference in phylogenetic distribution between gastric cancer and duodenal ulcer strains in the hpEurope group in Colombia. Sixty-six strains, with one exception, were classified as hpEurope irrespective of the *cagA* and *vacA* genotypes, and type of disease. STRUCTURE analysis revealed that Colombian hpEurope strains have a phylogenetic connection to Spanish strains.

**Conclusions:**

Our study showed that a phylogeographic origin determined by MLST was insufficient for distinguishing between gastric cancer and duodenal ulcer risk among hpEurope strains in the Andean region in Colombia. Our analysis also suggests that hpEurope strains in Colombia were primarily introduced by Spanish immigrants.

## Introduction

*Helicobacter pylori* is a spiral Gram-negative bacterium that infects more than half of the world’s population [1]. The transmission mechanism of *H. pylori* has not been fully clarified, but human-to-human spread via the oral-oral or fecal-oral routes is thought to be the most plausible [2]. *H. pylori* infection is now accepted to be linked to severe gastritis-associated diseases, including peptic ulcer and gastric cancer (GC) [1]. The infection remains latent in the majority of infected patients, and only a minority of individuals with *H. pylori* infection ever develop the disease [3]. Uemura et al. reported that GC developed in approximately 3% of *H. pylori*-infected patients during the observational period of 10 years, compared to none of the uninfected patients [4]. In addition to host, environmental, and dietary factors, the differences in the virulence of *H. pylori* strains are related to the varying outcomes of *H. pylori* infection. Virulence factors of *H. pylori*, such as *cagA, vacA, dupA, iceA, oipA,* and *babA*, have been shown to be predictors of severe clinical outcomes [5], [6]. Importantly, most of these virulence factors are associated with each other; *cagA*-positive strains also possess a *vacA* s1/m1 type and they are further closely linked to the presence of the *babA* and *oipA* “on” status [5].

The genetic diversity within *H. pylori* is greater than that of most other bacteria [7], and about 50-fold greater than that of the human population [8]. Furthermore, frequent recombination between different *H. pylori* strains [9] leads to only partial linkage disequilibrium between polymorphic loci, which provides additional information for population genetic analysis [10]. Recently, genomic diversity within *H. pylori* populations was examined by the multi-locus sequence typing (MLST) method using seven housekeeping genes (*atpA, efp, mutY, ppa, trpC, ureI,* and *yphC*) [10]–[12]. At present, seven population types have been identified based on geographical associations and designated as follows: hpEurope, hpEastAsia, hpAfrica1, hpAfrica2, hpAsia2, hpNEAfrica, and hpSahul [10]–[12]. hpEastAsia is common in *H. pylori* isolates from East Asia, and can be divided into the three subgroups hspEAsia, hspAmerind, and hspMaori. hpEurope includes almost all *H. pylori* strains isolated from ethnic Europeans, including people from countries colonized by Europeans. *H. pylori* is predicted to have spread from East Africa over the same time period as anatomically modern humans (∼58,000 years ago), and has remained intimately associated with their human hosts ever since [5], [11], [12].

The age standardized incidence rate (ASR) of GC in Colombia is reported to be relatively high (13.4/100,000 population) compared with other South American countries (average ASR = 10.3/100,000 population) (International Agency for Research on Cancer; GLOBOCAN2012, http://globocan.iarc.fr/). Notably, GC is more prevalent in the Colombian mountain region than on the coast [13], [14]. de Sablet et al. performed MLST to determine phylogeographic variation between mountain and coastal regions [15]. Interestingly, they found that all *cagA*-positive strains from GC high-risk regions belonged to hpEurope, whereas hpEurope and hpAfrica1 coexist in the GC low-risk regions [15]. In addition, subjects infected with hpEurope strains of *H. pylori* showed higher histopathological scores than those infected with hpAfrica1 strains. These observations suggest that the phylogeographic origin determined by MLST can be used as a predictor of GC risk.

However, these studies did not examine the phylogenetic origin according to clinical outcomes, and thus it remains unclear whether the phylogenetic origin is truly associated with clinical outcomes in Colombia. In this study, we examined the phylogenetic origin of the *H. pylori* strains from GC and duodenal ulcer (DU) patients living in Bogota, the capital and the largest city located in the Andean region in Colombia.

## Materials and Methods

### Patients

*H. pylori* strains were obtained from the gastric mucosa of *H. pylori*-infected Colombian patients with GC and DU who underwent endoscopy at Universidad Nacional de Colombia, Bogota, Colombia. GC and DU were identified by endoscopy, and GC was further confirmed by histopathology [16]. Patients with a history of partial gastric resection were excluded. Written informed consent was obtained from all the participants, and the protocol was approved by the Ethics Committee of Universidad Nacional de Colombia.

### Isolation and genotyping of *H. pylori*

Antral biopsy specimens were obtained for the isolation of *H. pylori* using standard culture methods as previously described [17]. *H. pylori* DNA was extracted from confluent plate cultures expanded from a single colony using a commercially available kit (QIAGEN, Valencia, CA). The status of *cagA* was determined by polymerase chain reaction (PCR) for the conserved region of *cagA* and for direct sequencing using the primer pair cagTF; 5′-ACC CTA GTC GGT AAT GGG-3′ and cagTR; 5′-GCT TTA GCT TCT GAY ACY GC-3′ (Y  =  C or T) designed in the 3′ repeat region of *cagA*, as described previously [18]. The PCR conditions were initial denaturation for 5 min at 95°C, 35 amplification steps (95°C for 30 s, 56°C for 30 s, and 72°C for 30 s), and a final extension cycle of 7 min at 72°C, using Blend Taq DNA polymerase (TOYOBO, Osaka, Japan). The absence of *cagA* was confirmed by the presence of *cagA* empty site, as previously described [19]. PCR products were purified with a QIAquick Purification Kit (QIAGEN) according to the manufacturer’s instructions. The amplified fragment was detected by electrophoresis in a 1.5% agarose gel that was subsequently stained with ethidium bromide and visualized using an ultraviolet trans-illuminator.

The *vacA* genotyping (s1, s2, m1, and m2) was performed as described previously [20], [21]. Primers for the signal region yielded a 259 bp and 286 bp fragment for s1 and s2 variants, respectively. Primers for the middle region yielded a 570 bp and 645 bp fragment for m1 and m2 variants, respectively.

### Phylogenetic analysis of *H. pylori* strains

MLST of the seven housekeeping genes (*atpA, efp, mutY, ppa, trpC, ureI, yphC*) was determined by PCR-based sequencing as described previously [7], [22]. Direct DNA sequencing was performed using an AB 3130 Genetic Analyzer (Applied Biosystems, Foster City, CA) according to the manufacturer’s instructions. For construction of the phylogenetic tree based on MLST genotypes, sequence datasets of the 7 housekeeping genes of hpAfrica2, hpAfrica1, hpNEAfrica, hpEurope, hpSahul, hpAsia2, hspMaori, hspAmerind, and hspEAsia strains (60, 181, 61, 566, 49, 17, 80, 18, and 177 strains, respectively, 1,209 strains in total) were obtained from the PubMLST database (http://pubmlst.org/). These sequence datasets were combined with our data from 66 Colombian strains. Neighbor joining trees were constructed by MEGA 5.0 using Kimura-2 parameters [23].

### Population structure analysis of *H. pylori* strains

We analyzed bacterial population structure using STRUCTURE (v.2.3.2) software [24]. Markov Chain Monte Carlo (MCMC) simulations of STRUCTURE were run in the admixture model with burn-in of 20,000, followed by 30,000 iterations for each run. The number of tentative populations (K) was set from 7 to 10 and 5 runs were executed for each K.

### Nucleotide sequence

Nucleotide sequence data reported here are available under the DDBJ accession numbers AB923031 to AB923492.

## Results

We included 35 GC patients and 31 DU patients. The strains isolated from GC patients included 24 *cagA*-positive and 11 *cagA*-negative. Twenty-nine were *vacA* s1m1 genotype and 6 were *vacA* s2m2 genotype. All 24 *cagA*-positive GC strains showed the *vacA* s1m1 genotype. For 31 strains from DU patients, 22 strains were *cagA*-positive and the remaining 9 were *cagA*-negative. The *vacA* s1m1 genotype was found in 16 strains, *vacA* s1m2 in 5 strains, and *vacA* s2m2 in 10 strains. Among 22 *cagA*-positive strains from DU patients, 14 strains possessed the *vacA* s1m1 genotype. Five strains were s1m2 and the remaining 3 strains were s2m2.

**Figure 1:**
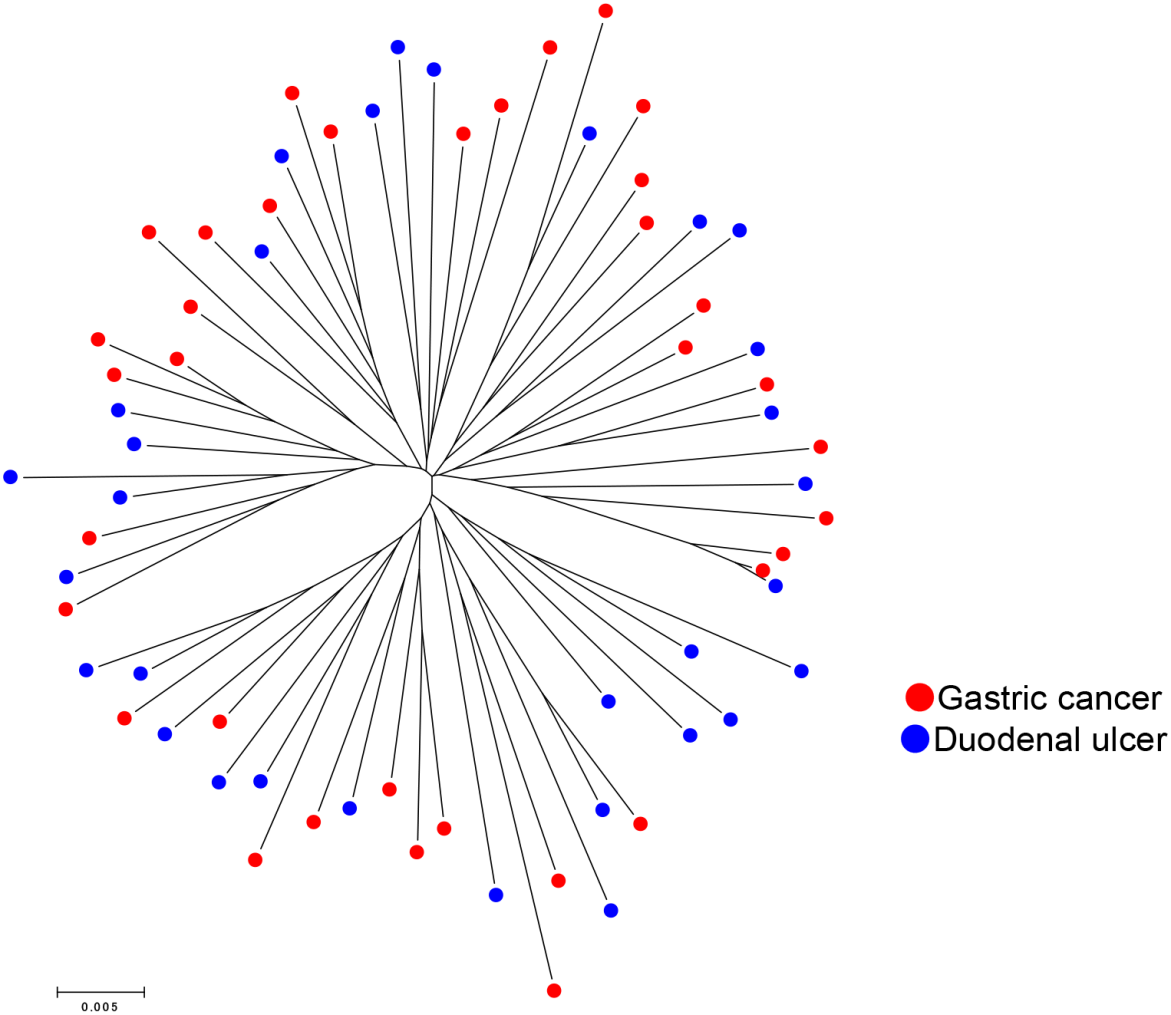
Phylogenetic tree generated based on the 7 housekeeping genes in Colombian strains of *H. pylori*. Sequence datasets of the 7 housekeeping genes of 66 Colombian strains were used to construct the tree. Red circles represent gastric cancer and blue circles represent duodenal ulcer strains. Neighbor joining trees were constructed in MEGA 5.0 using Kimura-2 parameters.

**Figure 2:**
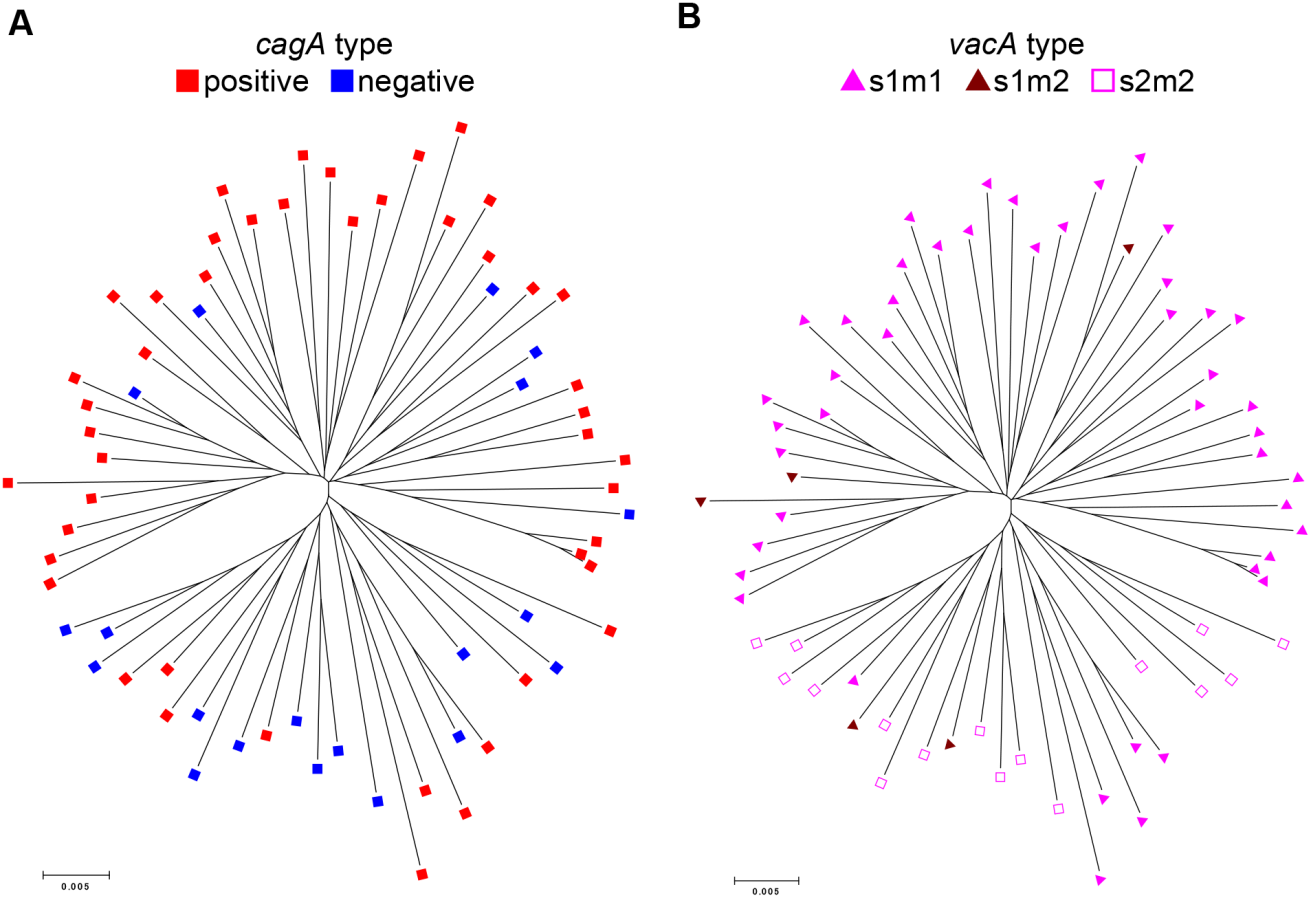
The status of *cagA* (a) and *vacA* (b) mapped on the MLST tree.

The population types of 35 strains isolated from patients with GC and 31 from DU were analyzed by MLST. The phylogenetic tree of 66 Colombian strains based on the MLST sequences and the types of disease are shown in Figure 1. GC and DU strains were scattered in the MLST tree; thus, we did not find any difference in phylogenetic distribution between GC and DU strains. Figure 2A shows the *cagA* status of the strains on the MLST tree. Strains from *cagA*-positive and *cagA*-negative could not be divided clearly, although *cagA*-negative strains were relatively clustered in the same branch. Figure 2B shows *vacA* types of the strains on the same MLST tree. Sixteen *vacA* s2m2 strains were clustered in a sub-branch together with two s1m2 and six s1m1 strains. The remaining three *vacA* s1m2 strains were located among the 39 *vacA* s1m1 strains.

**Figure 3:**
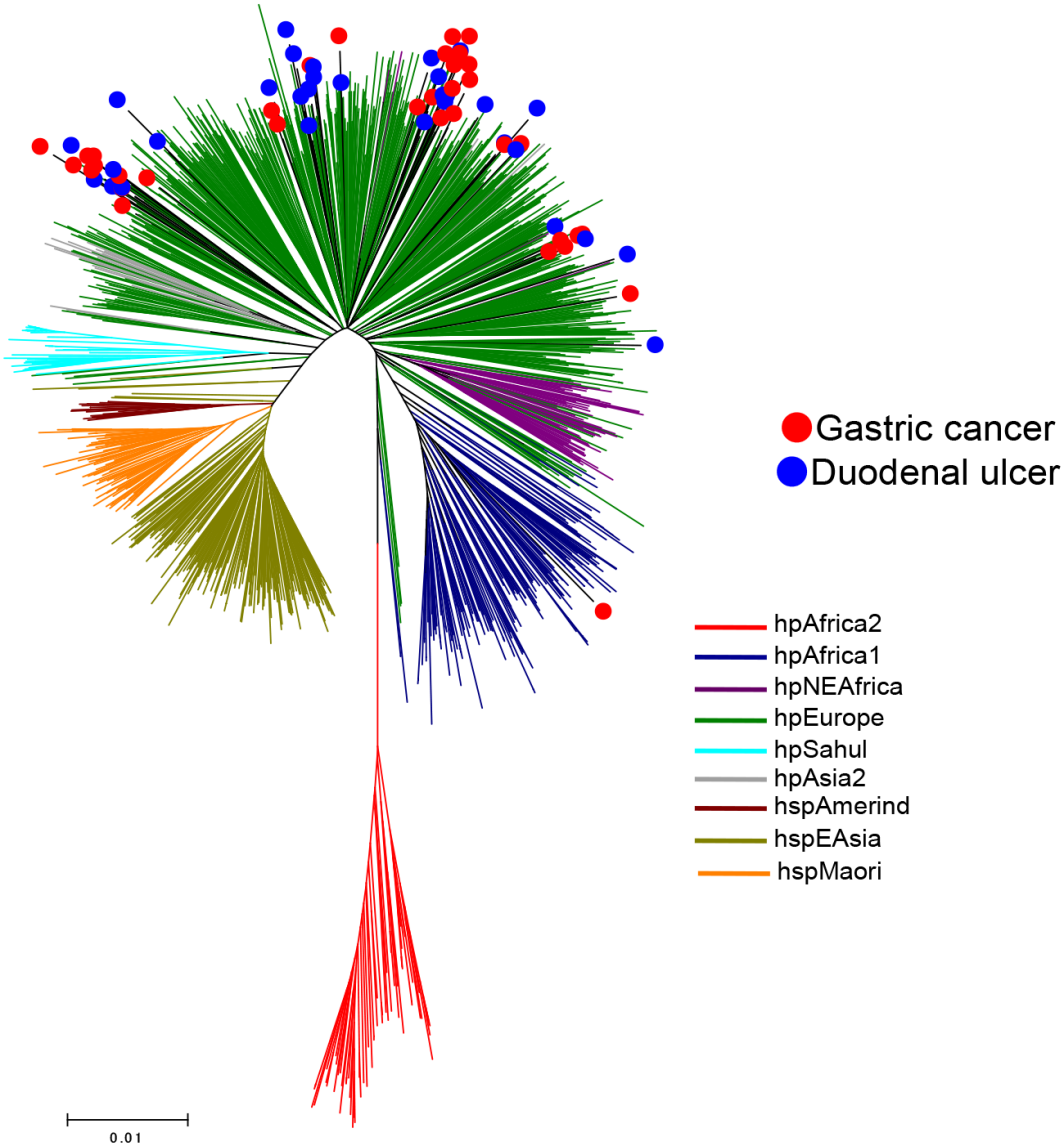
Phylogenetic tree of the 66 Colombian strains and 1,209 PubMLST strains. MLST sequence datasets of 1,209 strains were obtained from PubMLST database (60 strains of hpAfrica2, 181 of hpAfrica1, 61 of hpNEAfrica, 566 of hpEurope, 49 of hpSahul, 17 of hpAsia2, 80 of hspMaori, 18 of hspAmerind, and 177 of hspEAsia). The 66 Colombian strains that were sequenced are marked with red or blue circles according to the diseases.

Next, we constructed a phylogenetic tree based on MLST sequences of 66 Colombian strains and 1,209 reference strains of hpAfrica2, hpAfrica1, hpNEAfrica, hpEurope, hpSahul, hpAsia2, hspMaori, hspAmerind, and hspEAsia, deposited in pubMLST database (60, 181, 61, 566, 49, 17, 80, 18, and 177 strains, respectively) (Figure 3). Among the 66 Colombian strains, only one *cagA*-positive strain was located among the sub-branches of hpAfrica1. The remaining 65 strains were scattered among hpEurope sub-branches irrespective of the types of disease. Thus, no association was observed between the branching of the phylogenetic tree and clinical outcomes.

**Figure 4:**
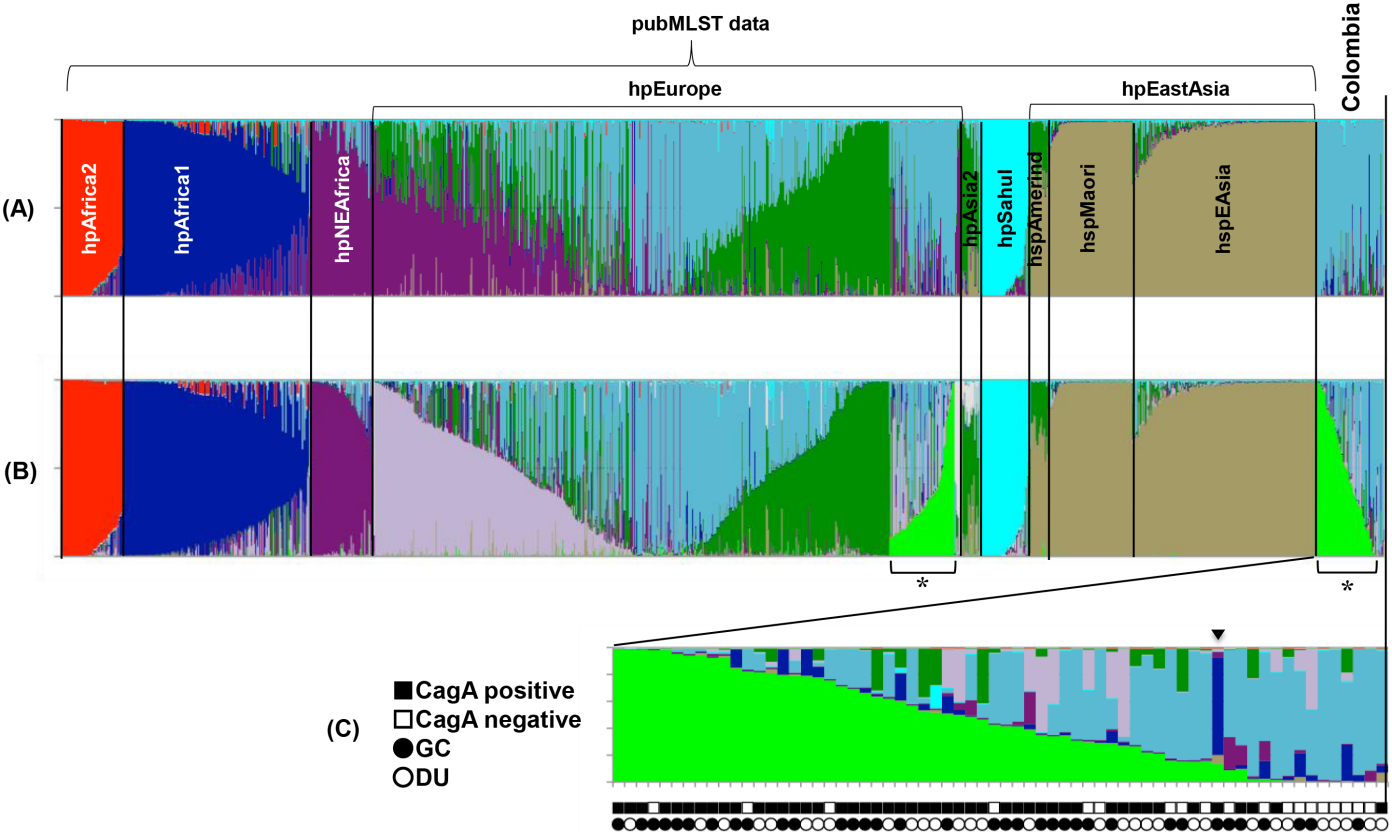
Population structure of Colombian strains. Results of population analysis by STRUCTURE (A) with K, or a tentative number of population, set to 7, (B) with K = 10, (C) magnified chart of Colombian strains. Each vertical bar represents one sample. Colors indicate population components and the lengths of the colors in a vertical bar are proportional to the probability that the sample belongs to the population of the color. The order of the samples is the same in all the bar charts. The stars in panel B represent Colombian strains and hpEurope strains that share the same population component. *cagA* types and diseases of Colombian strains are shown by marks below the bar chart (C).

To investigate the population structure of the Colombian strains, we performed population analysis using STRUCTURE software [24]. For this analysis, we used the same 1,257 strains (1,209 reference strains and 66 Colombian strains) that were used for the MLST phylogenetic analysis. STRUCTURE software performs MCMC simulation to classify individuals for a given number of populations (K). For a given K, STRUCTURE determines K population components and represents them by K colors using one color to represent one population component. We performed STRUCTURE analysis by setting K from 7 to 10 and executed simulations five times for each K. Figure 4 shows the results of K = 9 and 10 whose posterior probability is the best of the five runs (the most probable results). Each vertical line of the bar charts represents one strain and the colors of a line indicate populations to which the strain may belong. The lengths of the colors in a line are proportional to the probability that the strain belongs to each population. Because the hpEurope group is an admixture of multiple origins, this group shows complex colors.

In the result of K = 7, the Colombian strains showed common colors with hpEurope strains (Figure 4A). When K was set to 10, the Colombian and several hpEurope strains presented a different color from others that represents a population component specific to these strains (light green bars marked with stars in Figure 4B). This implies that these strains have basic similarity with European strains but are distinguishable from others if examined in detail. Figure 4C is a magnification of the Colombian strains. The bars were aligned from left to right in descending order of the light green component. *cagA* types and diseases are shown by marks below the bar chart. As this figure shows, *cagA*-negative strains were frequent on the right end. While most of the Colombian strains had a specific component and/or a common population component with hpEurope, one strain showed higher similarity with hpAfrica1 (Figure 4C, the bar marked with a triangle). This strain corresponds to the one that belongs to the hpAfrica1 sub-branch in the MLST phylogenetic tree (Figure 3).

In the data taken from PubMLST, we picked 63 hpEurope strains that contained the light green population component at 10% or higher and researched their sampling location. Twenty five strains were isolated from Colombia, although they were classified as hpEurope, 19 were from Spain, 4 were from Venezuela, and others are from various countries (Table S1).

## Discussion

Host, environmental, and dietary factors, and the differences in *H. pylori* strains are related to the varying outcomes of *H. pylori* infection. In general, the distribution of the incidence of GC is closely related to these *H. pylori* groups defined by population analysis based on MLST [25]. A high incidence of GC was found in the regions in which hpEastAsia strains prevail (especially hspEAsia). However, the incidence of GC is very low in Africa, where most strains are hpNEAfrica, hpAfrica1, or hpAfrica2, and in South Asia, where most strains are hpAsia2. Overall, the low incidence of gastric cancer in African and South Asian countries might be explained, at least in part, by the different genotypes of *H. pylori* circulating in different geographic areas. Intriguingly, a recent report on *cagA*-positive strains in Colombia showed that all strains from high-risk regions of GC belong to hpEurope, whereas hpEurope and hpAfrica1 strains coexist in the low-risk regions [15]. In addition, subjects infected with hpEurope strains of *H. pylori* showed higher histopathological scores than those infected with hpAfrica1 strains. The authors concluded that the difference in bacterial populations can be used as a predictor of GC risk.

In this study, we included *H. pylori* strains isolated from Colombian patients with GC and DU but not gastritis. Patients with only gastritis at the time of endoscopy may develop DU and GC later in life; conversely, DU patients rarely develop GC in their lifetime [4], [26]. Therefore, although *H. pylori* infection promotes the development of both GC and DU, GC and DU are considered the variant outcomes. In this study, we did not find any difference in phylogenetic groups between GC and DU strains. Our Colombian strains were found to belong to hpEurope, except for one hpAfrica1 strain. Although Colombian strains were divided into several sub-branches in the phylogenetic trees, there was no correlation between the branches and the diseases (Figures 1 and 3). Therefore, the topology of the MLST tree does not act as a marker to evaluate GC and DU risk for hpEurope strains in Colombia. In fact, even in the study conducted by de Sablet et al., all *cagA*-negative strains were also considered to belong to hpEurope [15]. The authors stated that subjects infected with *cagA*-negative strains had very low histopathological scores; therefore, hpEurope strains without the presence of *cagA* can be less virulent. In addition, we previously reported that strains with more than three repeat regions of the 3′ region in *cagA* were associated with significantly higher scores for gastric mucosal atrophy and intestinal metaplasia than those with fewer repeat regions in Colombia [20]. The prevalence of strains with more than three repeat regions was higher in GC than DU [20]. Thus, the *cagA* type rather than phylogeographic origin can be a better predictor of GC for hpEurope strains [27].

We previously found that although OipA and *cag* PAI are linked, only OipA was an independent risk factor for DU [28]. OipA in *cagA*-positive strains may contribute to phylogeographic variation determined by MLST analysis. In addition, we reported that *jhp0045* and *jhp0046* were significantly associated with GC in *cagA*-positive strains in Colombia [29]. Furthermore, we reported that infections with *dupA*-positive strains increased the risk for DU but were protective against GC in Colombia [30]. Therefore, the status of other virulence factors might be correlated with the phylogenetic tree obtained by performing MLST [5]. The relationships between the phylogeny of housekeeping genes and *cag* PAI phylogeny have been reported [31]. The phylogeny of most *cag* PAI genes was similar to that of MLST, indicating that *cag* PAI was probably acquired only once by *H. pylori*, and its genetic diversity reflects the isolation by distance which has shaped this bacterial species since modern humans migrated out of Africa [31]. In the present study, the phylogenetic tree based on MLST was not correlated with the type of *cagA* or *vacA*, although strains with *cagA*-negative or *vacA* s2m2 genotypes were relatively clustered. Only one strain belonged to hpAfrica1, and this strain possessed *cagA*, suggesting that *cagA* and *vacA* genotypes were not determining factors for phylogenetic origin based on MLST in the Andean region in Colombia. However, we previously reported that the *cagA* type was associated with a phylogenetic cluster in Okinawa, Japan [22], [27]. Strains from Okinawa were divided into 3 subpopulations and one admixture group influenced by Western strains. The phylogeographic topology and subpopulations were significantly associated with clinical outcomes; however, the difference in phylogeographic topology was due to the status of *cagA* (East-Asian type, Western-type *cagA*, and *cagA*-negative) and *vacA* (m1 or m2) of the subpopulations. GC was more prevalent in the cluster containing mostly East-Asian-type *cagA* strains. The results of MLST showed that the prevalence of GC in each group did not differ when only Western-type *cagA* strains or *cagA*-negative strains were used [27]. Thus, phylogeographic origin can act as confounding factor in predicting virulence factors but not disease outcome (e.g., in hspEAsia, most are *cagA*-positive, *vacA* s1/m1 type, *babA*-positive and *oipA* “on” status). In a recent study, not only *H. pylori* ancestry but also coevolution between human and *H. pylori* was found to be the best predictor for precancerous lesions [32]. Further studies are required to confirm the relationship between the ancestral origin of *H. pylori* irrespective of virulence factors and clinical outcomes.

In this study, we included the Mestizos patients mainly living in Bogota city, which is located in the mountain area (2,600 m above sea level). This area is generally populated by Mestizos, a mixed population who have descended from aboriginal Americans (Amerindians) and European people dating from the Spanish colonization period [33]. Amerindians are hypothesized to have been infected originally with hspAmerind strains that belong to the subpopulation of hpEastAsia [10]. Therefore, we expected that *H. pylori* isolated from Mestizos would display a mixed type of hpEurope and hspAmerind. Interestingly, all cases but one in our study were infected with hpEurope strains, consistent with a previous study [15]. STRUCTURE analysis revealed that several strains with hpEurope showed a mosaic pattern; however, they were not a mixture of hpEurope and hspAmerind. This observation supports the hypothesis that hpEurope strains possess a competitive advantage over indigenous hspAmerind strains as described previously [34], [35]. Surprisingly, our previous study showed that even in 16 strains isolated from Huitoto, a primitive, isolated group living in the Amazonian jungles in Colombia, 4 strains were infected with hspAmerind, while the remaining 12 were hpEurope strains [12]. In addition, we found that several Amerindian strains from Colombia possessed Western-type *cagA* but not East-Asian-type *cagA* [36]. It remains unclear why most Amerindian *H. pylori* strains have genotypes typical of strains from Western countries even in sites where Western influence appears to have been minimal. hpEurope, rather than hspAmerind strains, might be easily adapted to the environmental conditions and selective pressure exerted by the host. Two hypotheses, one based on strain competition and the other on strain subversion by transformation, were suggested as reasons for the displacement of hspAmerind by hpEurope [34]. Further study will be necessary to define the mechanism(s) responsible for the *in vivo* loss of the Amerindians strains when in competition with Old World strains. Interestingly, we found that several Colombian strains showed a specific population component, and this component was shared not only with other South American strains, but also with Spanish strains deposited in PubMLST. The city of Bogota, founded in 1538, became one of the principal administrative centers of the Spanish possessions in the New World (along with Lima and Mexico City) [33]. Our analysis suggests that hpEurope strains in Colombia were introduced mainly by Spanish immigrants. Population typing of *H. pylori* is a useful tool for mapping human migration patterns [37]; indeed, our findings might be useful to elucidate those of modern humans in South America. Although our MLST analysis based on seven housekeeping genes suggests that most of the Colombian strains were replaced by hpEurope strains, relics of ancestral aboriginal strains could remain in other parts of the genome. More extensive worldwide and genome-wide surveys will help us further understand the evolution and population structure of *H. pylori*.

One potential limitation of our study worth noting is that we could not obtain sufficient information about the ethnicity of the patients from whom the *H. pylori* strains were isolated. Indeed, it has been suggested that host ancestry might affect clinical outcomes as described above [32]. For example, inflammatory cytokine gene polymorphisms (*IL-1* gene cluster, *TNF-α*, *IL-10*, and *IL-8*) have been reported to be correlated with GC [38]–[43]. Furthermore, a family history of GC has been shown to contribute to the clinical outcomes [44]. In addition to other *H. pylori* virulence factors, this information may be useful for distinguishing between GC and DU risk. Further investigation is required to elucidate the association between *H. pylori* genotypes and disease outcomes.

## Conclusion

Our study revealed that a phylogeographic origin by MLST was not sufficient to distinguish between GC and DU risk in the Andean region in Colombia. A phylogenetic analysis including virulence factors of *H. pylori* might be necessary to more accurately determine clinical outcomes. Furthermore, our analysis also suggests that hpEurope strains in Colombia were introduced primarily by Spanish immigrants and their genotype was not influenced by Amerindians.

## Supporting Information

Table S1 (DOCX)
