# Supplementary material for: Helicobacter pylori from Gastric Cancer and Duodenal Ulcer Show Same Phylogeographic Origin in the Andean Region in Colombia
Source: PLoS One. 2014 Aug 14;9(8):e105392. doi: 10.1371/journal.pone.0105392 (PMC4133377; doi:10.1371/journal.pone.0105392)
Supplement: Table S1 — (DOCX) [file pone.0105392.s001.docx]

**Table S1**

| Country | #strain | ≥10% | Percentage |
| --- | --- | --- | --- |
| Colombia | 43 | 25 | 58.1 |
| Spain | 81 | 19 | 23.5 |
| Venezuela | 10 | 4 | 40.0 |
| Philippines | 13 | 3 | 23.1 |
| UK | 34 | 3 | 8.8 |
| Israel | 5 | 2 | 40.0 |
| Finland | 12 | 1 | 8.3 |
| France | 10 | 1 | 10.0 |
| Germany | 32 | 1 | 3.1 |
| Morocco | 7 | 1 | 14.3 |
| Peru | 4 | 1 | 25.0 |
| USA | 41 | 1 | 2.4 |
| Others | 917 | 1 | 0.1 |
| Overall | 1209 | 63 | 4.9 |

Note: The Country and #strain columns represent the sampling location and the number of strains obtained from PubMLST, respectively, and the column ≥10% represents the number of strains that contain the light green component 10% or higher in Figure 4(B), Percentage indicates the ratio of strains whose light green component is ≥10%.
